# The hidden *Heuchera*: How science Twitter uncovered a globally imperiled species in Pennsylvania, USA

**DOI:** 10.3897/phytokeys.96.23667

**Published:** 2018-04-17

**Authors:** Scott Schuette, Ryan A. Folk, Jason T. Cantley, Christopher T. Martine

**Affiliations:** 1 Pennsylvania Natural Heritage Program, Western Pennsylvania Conservancy, 800 Waterfront Drive, Pittsburgh, PA 15222, USA; 2 Florida Museum of Natural History, Dickinson Hall, University of Florida, Gainesville, FL 32611, USA; 3 Department of Biology, San Francisco State University, 1600 Holloway Ave. San Francisco, CA 94132, USA; 4 Department of Biology, Wayne E. Manning Herbarium, Bucknell University, 1 Dent Drive, Lewisburg, PA USA

**Keywords:** *Heuchera alba*, rare species conservation, Susquehanna River Valley, social media networks, iNaturalist, Twitter

## Abstract

The genus *Heuchera* is recognized as one of the most diverse endemic radiations of Saxifragaceae in North America, yet species delimitation and geographic distribution within the group remain controversial. Many species remain difficult to identify, including *Heuchera alba*, a narrow Appalachian endemic and globally imperiled (G2) taxon recorded only from West Virginia and Virginia that occurs in sympatry with *H. pubescens* and *H. americana*. A recent survey of the cliffside flora of the Shikellamy Bluffs, PA recorded dozens of *Heuchera* individuals that, through the use of social media, were positively identified as *H. alba*. Aided by examination of historical herbarium records, subsequent searches of similar habitats in Pennsylvania led to the discovery of seven more populations and established a significant range expansion for this rare species. The uncovering of *H. alba* in Pennsylvania is an exciting conservation outcome and an example of what can happen when botanists embrace a combination of modern and classical approaches to discovery and collaboration.

## Introduction

The genus *Heuchera* represents one of the most diverse endemic radiations of family Saxifragaceae in North America, with approximately 43 species (~10 in eastern North America). Its species are persistently difficult to identify (Wells and Shipes 2009), not least due to frequent introgressive hybridization throughout its history (reviewed in Folk et al. 2017). Despite substantial past taxonomic efforts, species delimitation and geographic distributions remain controversial and poorly resolved in this group, with the result that six species (14% of the genus) have been described in the last decade, in addition to several dramatic species re-circumscriptions (Folk and Freudenstein 2015, Folk and Alexander 2015).

*Heuchera alba* Rydb. (white alumroot, Saxifragaceae) was first described in 1926 based on a collection made in 1925 from Snowy Mountain in Pendleton County, West Virginia (Rydberg 1926). Since that time, both major monographs of *Heuchera* covering the eastern United States (Rosendahl et al. 1936; Wells 1984) reduced *H. alba* to synonymy with *H. pubescens* Pursh (1814), the only other large-flowered species known from the northern Appalachians. In the Flora of North America treatment of *Heuchera* (Wells and Shipes 2009), *H. alba* was again recognized at the species level, although no rationale for the shift in taxonomic concept was given. Recent morphological and molecular analyses of *Heuchera* (Folk and Freudenstein 2014, Folk et al. 2017, Folk and Stubbs in review) are consistent with this recent recognition of *H. alba* at the species level and its sister relationship to *H. pubescens*. To date, its documented range is restricted to high elevation acidic sandstone ridges and outcrops in the Ridge-and-Valley province of West Virginia and Virginia. Due to its narrow distribution and low number of populations, *Heuchera alba* is considered a globally imperiled (G2) species with 23 extant population occurrences (i.e. those observed since 1990), consisting of a total of 1500 individual plants throughout its entire range. Critically, fewer than 15 of the populations are considered viable and many of them are under threat from trampling and/or housing developments on high mountain ridges (NatureServe 2017).

In the summer of 2017, a group of scientists and students conducted a survey of the cliffside flora above the bank of the West Branch Susquehanna River in the Shikellamy Overlook area of Shikellamy State Park, just across the river from the town of Northumberland, PA. The primary goal of the survey was to assess the status of the golden corydalis (Corydalis aurea subsp. aurea Willd., Papaveraceae), a state-endangered species only known in Pennsylvania from this single site, as part of a new episode of the YouTube video series, “Plants are Cool, Too!” (Martine et al. 2018). During the course of the survey, numerous specimens were identified as *Heuchera americana* L. (American alumroot) using the Plants of Pennsylvania flora (Rhoads and Block 2007), a resource recognizing *H. americana* L. and *H. pubescens* as the only *Heuchera* species present in the state. A photo of one specimen was posted to Twitter, initiating an electronic discussion that led to a series of new collecting trips and establishment of the first confirmed state records in Pennsylvania for *Heuchera alba*. Here, we re-evaluate the resurrection of *H. alba* from synonymy and review variation in *Heuchera* species recorded beyond the borders of Pennsylvania to present conclusive evidence that *H. alba* should be added to the flora of the state. We discuss the extent of *H. alba* in Pennsylvania and the impact of this discovery on both our understanding of the overall distribution of this Appalachian endemic and its current conservation status.

## Methods

***Survey Sites.*** Visits to eight known *Heuchera* locations in the Ridge-and-Valley Ecoregion were made to collect fresh specimens for identification and determine population sizes. Based on herbarium label data and the habitat at Shikellamy State Park, a search image of potential suitable habitat was established to guide the surveys. GPS coordinates were recorded and geo-referenced images taken of the plants at each location. Using these data along with GPS data from annotated specimens, a map was generated in ArcGIS® Pro 2.0.1 (ESRI Redlands CA USA 2017) to illustrate the range expansion of *H. alba* into Pennsylvania. A general assessment of the habitat and site condition was performed to determine plant community associates and geological affinities.

***Taxonomic Identification.*** Representative specimens were collected from each of the visited locations and deposited at the Wayne E. Manning Herbarium at Bucknell University (BUPL) and the Carnegie Museum Natural History Herbarium (CM). These specimens were used to make species determinations. Specimens were identified using the Flora of North America treatment for *Heuchera*, the Flora of Virginia, and a taxonomic key for *Heuchera* that is soon to be published in the revision of the Gleason and Cronquist Manual of Vascular Plants of Northeastern United States and Adjacent Canada (Wells and Shipes 2009, Weakley et al. 2012, Folk and Stubbs in review). Consequent examinations of *Heuchera* specimens identified as *H. americana* and *H. pubescens*, housed in the Manning Herbarium, revealed four specimens of *Heuchera alba*, all collected in Union County between 1905 and 1949 from the cliffs at and around Shikellamy State Park.

***Conservation Rank Assessment.*** A standardized rank assessment method used by all heritage programs to assist with determining conservation statuses for species of concern is encapsulated in a rank calculator tool that analyzes populations at regional and global scales (NatureServe 2015). A population is defined as a collection of individuals of a species that is separated from the next collection of individuals by a minimum of 1 kilometer. An exception to this definition is those species that populate river corridors where the minimum separation distance is 10 kilometers. Using this definition, eight extant populations of *Heuchera alba* in Pennsylvania were analyzed to determine the state conservation status. The following parameters were used for the rank calculation. Range Extent: 5,000–20,000 km^2^, Area of Occupancy: 1 (4 km^2^ grid cell), Number of Occurrences: 6–20, Population Size: 250–1,000 individuals, Viability: 4–12 occurrences with good viability, Environmental Specificity: Narrow (specialist with key requirements), Threat Impact: Low.

## Results

Pennsylvania populations of *Heuchera alba* grow primarily on exposed rock outcrops on cliffs and in shale woodland and barrens plant communities. Populations are found on four different acidic sandstone geologies, including Burgoon Sandstone, the Catskill and Foreknobs formations, and the Hamilton Group of siltstone (Table 1). With the exception of the Burgoon sandstone, which is Mississippian, the other populations are all found over Devonian geology (Miles and Whitfield 2001). These different sandstones are considered to have acidic pH ranges.

**Table 1.** Survey locations with general habitat information for *Heuchera alba* in Pennsylvania.

County	Locality	Habitat	Geology
Bedford	Hopewell OutcropsWoy Bridge Barrens	Exposed, south-facing outcrops along road above Yellow CreekExposed, south-facing outcrops in shale woodland	Burgoon Sandstone (quartzitic sandstone)Catskill Formation (shale, mudstone, sandstone)
Huntingdon	Aitch Barrens/Raystown LakeHawn’s Overlook Barrens/Raystown Lake	Exposed, southwest-facing outcrops in shale woodlandExposed, west-facing outcrops in large shale barren	Foreknobs Formation (sandstone, siltstone, mudstone, shale)Foreknobs Formation (sandstone, siltstone, mudstone, shale)
Perry	Cliffs at Montgomery’s Ferry	Exposed east/northeast-facing cliff above the Susquehanna River	Hamilton Group (siltstone, claystone, sandstone, shale)
Snyder	Cliffs at Dundore	Exposed east-facing cliff above the Susquehanna River	Catskill Formation/ Sherman Creek Member (sandstone, siltstone, claystone)
Union	Shikellamy Bluffs/Shikellamy State ParkCliffs at Gundy’s Farm	Exposed east/northeast-facing cliff above West Branch Susquehanna RiverExposed east/northeast-facing cliff above West Branch Susquehanna River	Catskill Formation (shale, mudstone, sandstone)Catskill Formation (shale, mudstone, sandstone)

**Figure 1.**
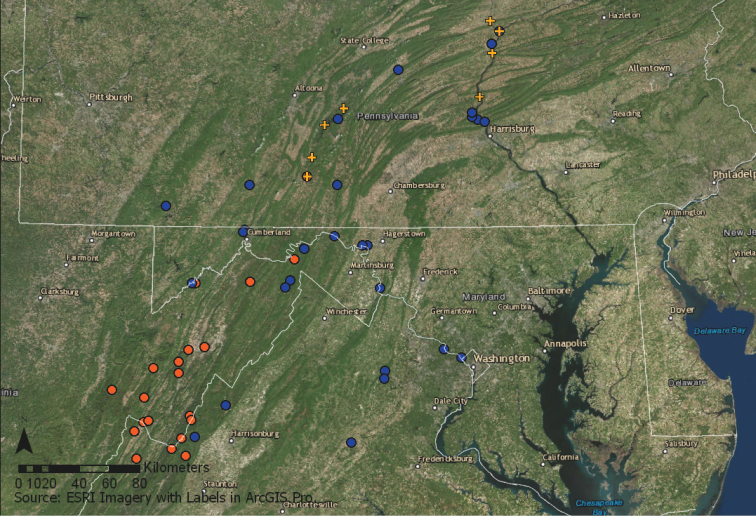
Range extent of *Heuchera alba* and *Heuchera pubescens* in the Appalachian Ridge and Valley. Orange circles indicate range and location of *H. alba* in WV and VA; Blue circles indicate partial range and location of *H. pubescens*; Orange crosses represent new locations of *H. alba* in Pennsylvania.

The eight extant populations of *H. alba* total between 250–1,000 individuals and range widely from northeastern Bedford County to southeastern Union County, but occupy <4 km^2^ over that range extent in Pennsylvania (Figure 1). These populations extend the known range for *H. alba* approximately 200km northward in the Appalachian Ridge and Valley Ecoregion (Figure 1) The population at Shikellamy State Park is by far the most robust with between 400–800 individuals over a 0.7 km^2^ area, while the remaining populations have fewer than 50 individuals. The Gundy’s Farm and Woy Bridge populations have fewer than 15 individuals. All factors, including potential threats, together yield a rank of critically imperiled at the state level (S1). However, uncertainty about the number of misidentified specimens in herbaria and the amount of unexplored available habitat in Pennsylvania suggests that a range rank of critically imperiled to imperiled (S1S2) is the more appropriate rank (Table 2).

**Table 2.** NatureServe Rank Calculator assessment output with comments concerning population number, size, and area of occupancy of *Heuchera alba* in Pennsylvania.

	Rank Calculator Categories and Values for Pennsylvania Populations		Comments
**RARITY**	Range Extent	E = 5,000 to 20,000 km^2^	Relatively wide range extent throughout the Ridge and Valley province in Pennsylvania
	Area of Occupancy (AOO): 4 km^2^ grid cells	A = (1) 4 km^2^ grid cells	Very small AOO with a total of < 1 km^2^ throughout the PA range
	Number of Occurrences	B = 6 to 20	Currently 8 confirmed populations
	Population Size	C – 250 to 1000 individuals	Total number combined from all occurrences in Pennsylvania
	Good Viability/Ecological Integrity: # of occurrences	C = Few (4-12) occurrences with excellent viability or ecological integrity	A small number of occurrences have large population sizes and are in hard to access habitats
	Environmental specificity	B = Narrow (Specialist)	Prefers acidic rocky outcrops, cliffs, and exposed rocky woodlands
**THREATS**	Assigned Overall Threat Impact	D = Low	Overall a relatively low threat impact except for the few populations that are along roadsides
**TRENDS**	Short Term Trend	N/A	Not enough data to assign trend
	Long Term Trend	N/A	Not enough data to assign trend
**Calculated Rank**: **S1**; **Assigned Rank**: **S1S2; Assigned Rank Reasons**: Given the extensive available potential habitat in Pennsylvania further survey work in addition to in-depth herbarium studies are needed to determine full range extent and area of occupancy necessary for more confident ranking. Until then a range rank of S1S2 is recommended for this newly reported species to Pennsylvania.			

## Discussion

**Figure 2.**
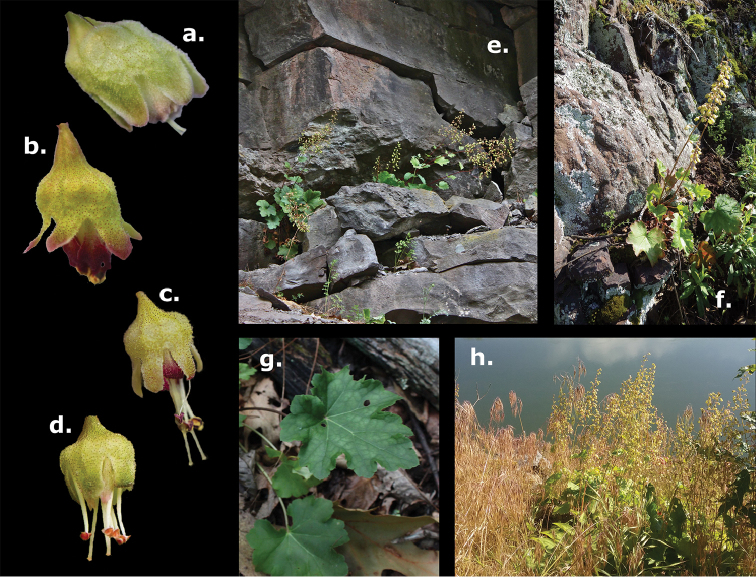
Images **a–d** Flowers of four *Heuchera* taxa overlapping in range in PA, WV, and VA. Flowers are shown at the original angle on the inflorescence; with the exception of the wild accession in (**d**), all flowers were obtained under common greenhouse conditions after at least a year of cultivation **a** *H. alba* (North Fork Mountain, WV; Folk 63 [deposited at OS]) **b** *H. pubescens* (Pilot Mountain, NC; Folk 96 [deposited at OS]) **c** *Heuchera × hispida* (=H. americana var. hispida; near Sandstone Falls, WV; Folk 104 [deposited at OS]) **d** H. americana var. americana (Blue Ridge Parkway at Twenty Minute Cliff, VA; Folk 102 [deposited at OS]). Images **e–h** *H. alba* plants growing at North Fork Mountain, WV (**e, g**) and Shikellamy State Park, PA (**f, h**). All photos R. Folk, except (**f**) and (**h**) by C. Martine.

**Figure 3.**
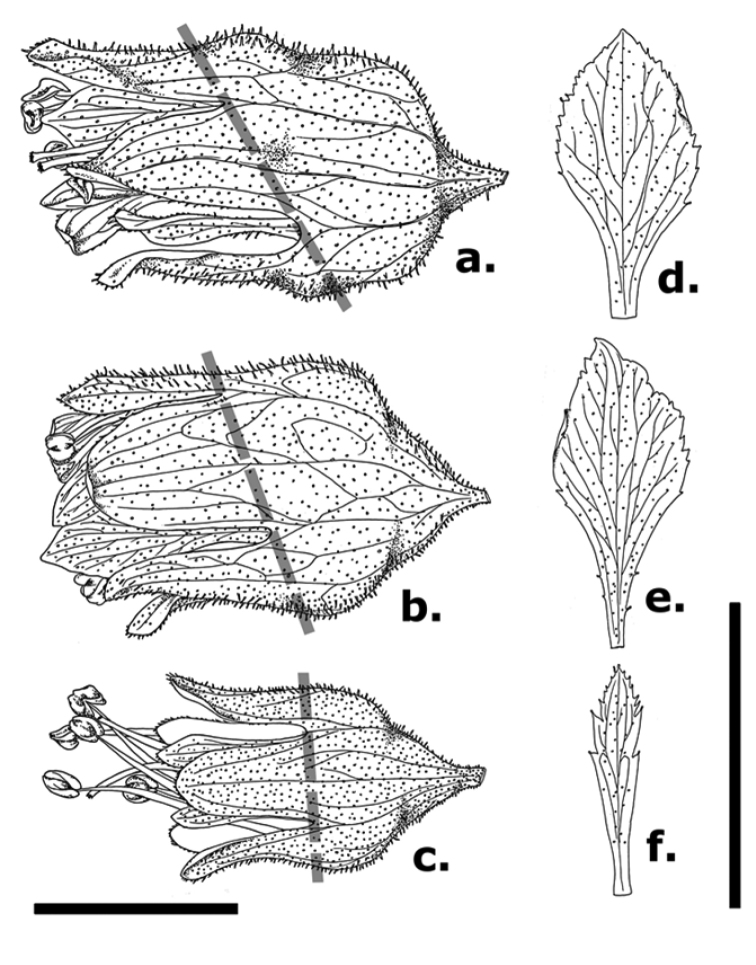
Comparisons of flowers and single petals (adaxial surface) of three *Heuchera* species co-occurring in PA, WV, and VA, based on spirit-preserved materials: *Heuchera alba* (**a, d** North Fork Mountain, WV; Folk 63 [deposited at OS]), *Heuchera pubescens* (**b, e** Rocky Knob Overlook, Blue Ridge Parkway, VA; Folk 100 [deposited at OS]), and unhybridized *H. americana* (i.e. H. americana var. americana **c, f** Chestoa Overlook, Blue Ridge Parkway; Folk 92 [deposited at OS]). Dotted gray lines show degree of hypanthium zygomorphy. Both scale bars represent 5 mm; the left-hand scale bar applies to **a–c** the right-hand bar is for **d–f**. Illustrations by R. Folk

The Plants of Pennsylvania flora (Rhoads and Block 2007) currently recognizes two species of *Heuchera*, *H. americana* and *H. pubescens*, as occurring within the state. *Heuchera alba* should now be considered a third member of the genus for Pennsylvania. The species differs from *H. americana* in the length of stamen exertion, length of the free hypanthium, and individual flower shape; and is distinct from *H. pubescens* in flower size, color, and aspect on the inflorescence (see Figs 2, 3). Floral aspect, in particular, is absent from most recent taxonomic treatments and has only recently been recognized as variable and taxonomically informative in *Heuchera* (Folk and Freudenstein 2014); in *H. alba*, flower orientation is subhorizontal, whereas in *H. pubescens* flowers are more or less completely pendent (Folk and Stubbs in review). *Heuchera alba* is distinct from both *H. americana* and *H. pubescens* in having greater floral zygomorphy (Fig. 3).

The “discovery” of *Heuchera alba* in Pennsylvania is especially illustrative as a case study for the potential benefits of merging classical botanical studies with modern modes of information sharing, particularly non-traditional scientific communication through the use of social media networks. The identification of *H. alba* at Shikellamy State Park was only incidentally facilitated because C. Martine (@MartineBotany) posted an image of a specimen on Twitter (Martine 2017) while filming part of the YouTube science outreach program, “Plants are Cool, Too!” (Martine et al. 2018). Within hours of the initial Tweet going live, R. Folk (@ry_folk) had replied to the post, suggesting that what had been identified as *H. americana* was probably *H. alba* – an inference made with confidence stemming, in part, from the fact that S. Schuette (@mossman2000) had recently posted a likely *H. alba* image from another Pennsylvania locality on iNaturalist.org (https://www.inaturalist.org/observations/2825610). Unequivocal support for the identification required the collection and examination of specimens, however, including inspection of sheets held at the Wayne E. Manning Herbarium (BUPL) at Bucknell University. BUPL holds a handful of records for *H. alba* that had until now been identified as either *H. americana* or *H. pubescens*, including a specimen collected at the Shikellamy Bluffs by W. Manning in 1946 and another collected by a student ca. 1905 from a site (then known as “Gundy’s Farm”) just about a half-mile from the Bucknell campus. Importantly, the knowledge gleaned from the new and historical collections coupled with statewide bedrock maps (Berg et al. 1980) allowed the authors to make predictions about additional cliffside habitats in the region where *H. alba* might occur, leading to many of the new geographic records shown in Figure 1. Additional herbarium work at CM and PH is necessary to determine the number of misidentified specimens that may point to new locations for this imperiled species in Pennsylvania.

This discovery may also serve as a cautionary tale of relying entirely for plant identification on floras which, through no fault of their own, become incomplete or ‘static’ over time (both relative to taxonomic circumscriptions and also taxon distributions) and have the mixed benefit / danger of including only the species known at their time of writing to be in the geographic area of the flora. While the exclusion of species known from nearby (but outside the area covered) simplifies keys and makes identification easier, it can also decrease the likelihood of the discovery of range extensions.

The likelihood of additional populations of *H. alba* existing in Pennsylvania is quite high, but confusion between this species and the state’s other two might hamper the ease of discovery. Until the new treatment of *Heuchera* is published in the revision of the Gleason and Cronquist Manual of Vascular Plants of Northeastern United States and Adjacent Canada, using the identification key for *Heuchera* in the Flora of Virginia (Weakley et al. 2012) or the FloraQuest app (Weakley and Lee 2018) will facilitate quick determinations of species within the genus. We present a set of illustrations and photographs (Figs 2, 3) comparing *H. alba*, *H. americana*, and *H. pubescens*, plus the occasional hybrid between the latter two species (Heuchera × hispida Pursh.). Although the distributions of these taxa overlap in Pennsylvania, West Virginia, and Virginia, there are clear differences in substrate specificity between *H. alba* and the other two species. Acidic rock outcrops are the preferred habitat and substrate for *H. alba*, while *H. americana* grows in rich woods over base-rich granite and gneiss or shallow, rocky soils, and *H. pubescens* grows on circumneutral rock outcrops, ledges, and rock cuts (Wells and Shipes 2009). Local sympatry is rare across the range of these species, yet sympatric populations of *H. alba* and *H. pubescens* at the Shikellamy Bluffs location, together with the weak reproductive barriers present in this group (Wells 1979), raise questions about the potential for hybridization and the subsequent impacts to the population genetics of both species at this key site.

The preponderance of *H. alba* localities now recorded for Pennsylvania underlines the need to continually assess imperiled taxa with integrative field surveys and taxonomic methods, and might further suggest that the species is not quite as globally rare as previously understood. This assumption should be made with caution, however, based on two observations: 1) The Pennsylvania populations are all relatively small (see above) and restricted to specific and uncommon habitat conditions, and 2) Most populations suffer from incursions of exotic invasive species. For example, the “Gundy’s Farm” population mentioned above consists of just 12 individuals on a low cliff face inundated with *Lonicera morrowii* A. Gray, *Alliaria petiolata* (M.Bieb.) Cavara & Grande, *Celastrus orbiculatus* Thunb., and other invaders. While it consists of many more individuals, the Shikellamy Bluffs population also suffers from invasion – largely because mowing and weeding of the parkland above it consistently sends weedy debris and seeds over the edge of the cliff. This latter locality is particularly critical to the status of *H. alba* in Pennsylvania given its size and status as a protected area; and preservation of the population there was made more certain following recent efforts to nearly double the amount of protected bluffs habitat led by the Merrill W. Linn Land and Waterways Conservancy.

## Conclusion

The conservation needs for *Heuchera alba* will become more apparent as new population locations are revealed following a thorough review of existing *Heuchera* collections from the Ridge-and-Valley region of Pennsylvania. In the meantime, the Twitter-fueled identification of this species in Pennsylvania is an exciting outcome that provides a model for the sorts of strides we can make when botanists embrace a combination of modern and classical approaches to discovery and collaboration.
